# Nanoparticle inhalation augments particle-dependent systemic microvascular dysfunction

**DOI:** 10.1186/1743-8977-5-1

**Published:** 2008-02-12

**Authors:** Timothy R Nurkiewicz, Dale W Porter, Ann F Hubbs, Jared L Cumpston, Bean T Chen, David G Frazer, Vincent Castranova

**Affiliations:** 1 Center for Interdisciplinary Research in Cardiovascular Sciences, West Virginia University School of Medicine, Morgantown, WV, USA; 2 Department of Physiology and Pharmacology, West Virginia University School of Medicine, Morgantown, WV, USA; 3 Pathology and Physiology Research Branch, Health Effects Laboratory Division, National Institute for Occupational Safety and Health, Morgantown, WV, USA

## Abstract

**Background:**

We have shown that pulmonary exposure to fine particulate matter (PM) impairs endothelium dependent dilation in systemic arterioles. Ultrafine PM has been suggested to be inherently more toxic by virtue of its increased surface area. The purpose of this study was to determine if ultrafine PM (or nanoparticle) inhalation produces greater microvascular dysfunction than fine PM. Rats were exposed to fine or ultrafine TiO_2 _aerosols (primary particle diameters of ~1 μm and ~21 nm, respectively) at concentrations which do not alter bronchoalveolar lavage markers of pulmonary inflammation or lung damage.

**Results:**

By histopathologic evaluation, no significant inflammatory changes were seen in the lung. However, particle-containing macrophages were frequently seen in intimate contact with the alveolar wall. The spinotrapezius muscle was prepared for in vivo microscopy 24 hours after inhalation exposures. Intraluminal infusion of the Ca^2+ ^ionophore A23187 was used to evaluate endothelium-dependent arteriolar dilation. In control rats, A23187 infusion produced dose-dependent arteriolar dilations. In rats exposed to fine TiO_2_, A23187 infusion elicited vasodilations that were blunted in proportion to pulmonary particle deposition. In rats exposed to ultrafine TiO_2_, A23187 infusion produced arteriolar constrictions or significantly impaired vasodilator responses as compared to the responses observed in control rats or those exposed to a similar pulmonary load of fine particles.

**Conclusion:**

These observations suggest that at equivalent pulmonary loads, as compared to fine TiO_2_, ultrafine TiO_2 _inhalation produces greater remote microvascular dysfunction.

## Background

The association between cardiovascular disease and exposure to airborne particulate matter (PM) is well established. Nanotechnology is already well-integrated into our daily lives, and appears to offer humankind novel benefits limited only by our creativity. While the scope of these benefits is essentially unrestricted, benefits that are specific to human health include: molecular imaging/diagnosis, drug delivery, anticancer therapy and gene therapy. However, if the unrestricted use of nanotechnology is rushed to societal integration, and the health effects of nanoparticles are not clearly identified, such technology runs the risk of causing unanticipated adverse health effects [1], and the true potential of the technology will never be fully realized [2]. A fundamental first step in avoiding these possible outcomes is the realization that nanoparticles are not merely smaller variants of similarly composed fine particles. This is evidenced by the observation that nanoparticles are primarily manufactured to produce specific differences in surface reactivity, solubility or conductance.

Titanium dioxide ultrafine particles that fall within the nanoparticle size range (one dimension less than 100 nm) are commonly used as photocatalysts to clean air and water [3], as antibacterial agents on glass and steel [4], and as components of many cosmetics and sunscreens. Once in the lung, TiO_2 _nanoparticles initiate pulmonary responses such as airway inflammation [5], alveolar macrophage recruitment [6], and the activation of various growth factors and chemokines [7]. Ultrafine particles deposited in the lung may have the ability to translocate to systemic sites within 24 hours of deposition [8-10]. Similarly, within 24 hours of deposition a substantial portion of inhaled TiO_2 _nanoparticles escape phagocytosis and enter the alveolar interstitium [11] and pulmonary capillaries [12].

While the pulmonary responses to nano-sized titania particles appear to be well-understood, the systemic microvascular effects of exposure to such particles are unknown. We have previously shown that pulmonary exposure to fine PM (PM_2.5_, particles with a mean aerodynamic diameter of 0.1–2.5 μm) causes systemic microvascular dysfunction, most evident in the form of impaired or abolished endothelium-dependent arteriolar dilation [13,14]. These studies also provided evidence that this systemic effect is not necessarily the result of the inherent pulmonary toxicity of the particles, in that equivalent doses of titanium dioxide (TiO_2_) and residual oil fly ash caused similar, dose-dependent degrees of microvascular dysfunction. While this indicates that larger TiO_2 _particles elicit potent systemic microvascular effects, it cannot be used to conclude that ultrafine TiO_2 _exposure will produce qualitatively or quantitatively similar biologic effects.

Therefore, the purpose of this study was to characterize systemic microvascular function after pulmonary exposure (via inhalation) to fine or ultrafine TiO_2 _aerosols over a range of concentrations, and determine if ultrafine TiO_2 _particles are inherently more toxic than larger, fine TiO_2 _particles.

## Methods

### Experimental Animals

Specific pathogen free male Sprague Dawley [Hla:(SD)CVF] rats (6–7 wks old) were purchased from Hilltop Laboratories (Scottdale, PA) and housed in an AAALAC approved animal facility at the National Institute for Occupational Safety and Health. Rats were housed in laminar flow cages under controlled temperature and humidity conditions and a 12 hr light/12 hr dark cycle. Food and water were provided ad libitum. Rats were acclimated for 5 days before use and certified free of endogenous viral pathogens, parasites, mycoplasms, *Helicobacter *and CAR bacillus. To ensure that all methods were performed humanely and with regard for alleviation of suffering, all experimental procedures were approved by the Animal Care and Use Committees of the National Institute for Occupational Safety and Health, and West Virginia University.

### Inhalation Exposure

**Figure 1:**
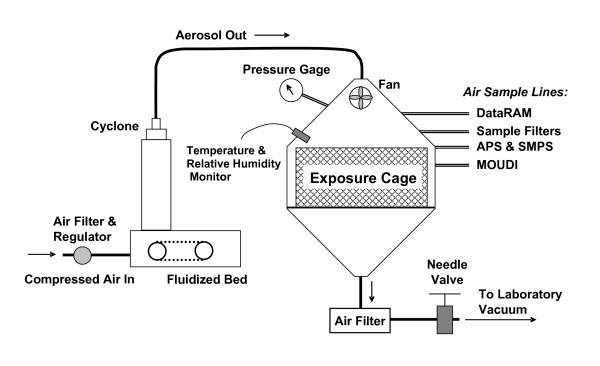
**Schematic diagram of the TiO_2 _inhalation exposure system.** Data-logging Real-time Aerosol Monitor (DataRAM). Aerodynamic Particle Sizer (APS). Scanning Mobility Particle Sizer (SMPS). Micro-Orifice Uniform Deposit Impactors (MOUDI).

An inhalation exposure system, containing a fluidized-bed powder generator, an animal chamber, and several aerosol monitoring devices, was developed for continuous generation and monitoring of ultrafine or fine TiO_2 _aerosols for rodent exposure [15]. A schematic of the system is presented in Figure 1. The system was designed based on the criteria of simplicity, ability to disperse fine/ultrafine TiO_2 _aerosols, and ease of maintenance. The ultrafine and fine TiO_2 _powders were obtained from DeGussa (Aeroxide TiO_2_, P25, primary particle size 21 nm, Parsippany, NJ) and Sigma-Aldrich (titanium (IV) oxide, 224227, primary particle size 1 μm, St. Louis, MO), respectively. To reduce the potential formation of agglomerates due to van der Waals force, the TiO_2 _powders were carefully prepared for generation by sieving (to remove the large agglomerates), drying (to avoid agglomerate formation due to high humidity), and storage (to prevent agglomerate attraction through contact charges). A fluidized-bed aerosol generator was used in this study because it was able to disperse powders effectively. A 19-liter metabolism chamber that contains an animal cage was modified for use as the whole-body exposure chamber. The cage can accommodate 3 rats for each exposure. During exposure, TiO_2_mass concentrations were continuously monitored with a Data RAM (DR-40000 Thermo Electron Co, Franklin, MA) and gravimetrically measured with Teflon filters. Aerosol concentrations between 1.5 and 20 mg/m^3 ^were achieved by adjusting the powder feed rate in the generator. Pulmonary deposition was estimated by the formula: Pulmonary Load = aerosol concentration × minute ventilation × exposure duration × deposition fraction, where minute ventilation and deposition fraction were estimated to be 200 cc and 10%, respectively. The deposition fraction of 10% was based upon Kreyling's alveolar deposition curve for inhaled ultrafine particles in the rat [16]. The particle size distributions of TiO_2 _aerosols were measured using a cascade impactor (MOUDI, MSP Co., Shoreview, MN), an electrical mobility classifier (SMPS, TSI Inc., Shoreview, MN), and an aerodynamic sizing instrument (APS, TSI Inc.). The impactor was used for measuring mass-based aerodynamic size distributions, while the latter two sizing devices were combined for determining number-based mobility size distributions. In addition, temperature, relative humidity, and pressure in the chamber were monitored throughout the exposure.

To verify that containment in the exposure chamber did not cause an unintended biologic response, Sham/Control exposures (that matched the exposure duration of the experimental groups) were performed throughout the course of these experiments. Because no discernable systemic or microvascular effect was observed in any condition, the data from these experiments were combined into a single "Sham/Control" group. Furthermore, the systemic and microvascular responses of this "Sham/Control" group were not different from those observed in naive rats (data not shown).

### Histopathology

At 24 hr post-exposure, lungs were rapidly removed and inflated with 6 ml 10% neutral buffered formalin, routinely processed overnight in an automated tissue processor, embedded in paraffin, sectioned at 5 μm, and stained with hematoxylin and eosin. The left lung lobe was evaluated by a board-certified veterinary pathologist in a blinded fashion.

### Measurement of Pulmonary Particle Deposition

At 24 hr post-exposure, lungs were removed, weighed, frozen at -80°C, and then lyophilized. After lyophilization, the samples were prepared for analysis by inductively-coupled plasma-atomic emission spectroscopy (ICP-AES) [17]. Lung TiO_2 _burden was expressed as total lung burden (μg).

### Intravital Microscopy

The spinotrapezius muscle preparation has been used for over 30 years as an experimental model to evaluate physiological and pathophysiological phenomena at the microvascular level [18,19]. The preparation has been an essential tool in the fundamental understanding of: capillary network development [20], neurogenic, humoral and myogenic control of microvascular resistance [21-23] and the physiological roles of O_2_, NO and Ca^2+ ^in the microcirculation [24-26]. Furthermore, the spinotrapezius muscle preparation has been continuously used to characterize pathophysiological microvascular consequences of chronic diseases such as diabetes [27], hypertension [23,28,29], and heart failure [27]. Arterioles of the first branching order were studied because these vessels, in conjunction with their upstream feed arteries, account for approximately 60% of total spinotrapezius muscle vascular resistance, and therefore, are of major importance for local blood flow regulation [28].

At 24 hr post-exposure, rats were anesthetized with sodium thiopental (Pentothal, 100 mg/kg, i.p.) and placed on a heating pad to maintain a 37°C rectal temperature. The trachea was intubated to ensure a patent airway, and the right carotid artery was cannulated to measure arterial pressure. The right spinotrapezius muscle was then exteriorized for microscopic observation, leaving its innervation and all feed vessels intact. After exteriorization, the muscle was gently secured over an optical pedestal at its in situ length. The muscle was next enclosed in a tissue bath for transillumination and observation. Throughout the surgery and subsequent experimental period, the muscle was continuously superfused with an electrolyte solution (119 mM NaCl, 25 mM NaHCO_3_, 6 mM KCl and 3.6 mM CaCl_2_), warmed to 35°C, and equilibrated with 95% N_2 _- 5% CO_2 _(pH = 7.35–7.40). Superfusate flow rate was maintained at 4–6 ml/min to minimize equilibration with atmospheric oxygen [30].

The animal preparation was then transferred to the stage of an intravital microscope coupled to a CCD video camera. Observations were made with a 20X water immersion objective (final video image magnification = 1460X). Video images were displayed on a high-resolution color video monitor and videotaped for off-line analysis. During videotape replay, arteriolar inner diameters were measured with a video caliper (Cardiovascular Research Institute, Texas A&M University).

Arteriolar endothelium-dependent dilation was evaluated by assessing the capacity for Ca^2+^-dependent endothelial NO formation in response to intraluminal infusion of the calcium ionophore A23187 (Sigma). Glass micropipettes were filled with a 10^-7 ^M solution of A23187, inserted into the arteriolar lumen, and A23187 was then infused directly into the flow stream for 2-minute periods at ejection pressures of 5, 10, 20 and 40 psi. A 2-minute recovery period followed each ejection. We have previously verified that this technique induces vasoactive responses that are not the result of mechanical artifact [13,31]. At the end of all intravital experiments, adenosine (ADO) was added to the superfusate (10^-4 ^M final concentration) to fully dilate the microvascular network and determine the passive diameter of each arteriole studied.

### Data and Statistical Analyses

Arteriolar diameter (D, μm) was sampled at 10-second intervals during all control and infusion periods. Resting vascular tone was calculated for each vessel as follows: Tone = [(D_pass _- D_c_)/D_pass_] × 100, where D_pass _is passive diameter under ADO and D_c _is the diameter measured during the control period (resting diameter). A tone of 100% represents complete vessel closure, whereas 0% represents the passive state. In order to evaluate arteriolar responsiveness between individual groups with subtle differences in resting diameter, arteriolar diameter was normalized. In this case, arteriolar diameter was expressed as a percentage of the maximum response and was calculated for each vessel as follows: Diameter (% of Maximum Response) = [(D_SS _- D_c_)/(D_pass_- D_c_)] × 100, where D_SS _is the steady state diameter achieved during A23187 infusion. All data are reported as means ± SE, where "n" represents the number of arterioles evaluated and "N" represents the number of rats studied. This distinction was made because the experimental unit in the current study was the arteriole. One to three microvessels were studied per rat. Given the inherent microvascular heterogeneity in any given tissue [32], judicious sampling of multiple vessels per rat serves not only to properly represent such heterogeneity, but also to ultimately reduce the number of animals required to complete the data sets. Statistical analysis was performed by commercially available software (Sigmastat, Jandel Scientific). One-way repeated measures ANOVA was used to determine the effect of a treatment within a group, or differences among groups. Two-way repeated measures ANOVA was used to determine the effects of group, treatment and group-treatment interactions on measured variables. For all ANOVA procedures, the Student-Newman-Keuls method for post hoc analysis was used to isolate pair wise differences among specific groups. Significance was assessed at the 95% confidence level (P < 0.05) for all tests.

## Results

**Figure 2:**
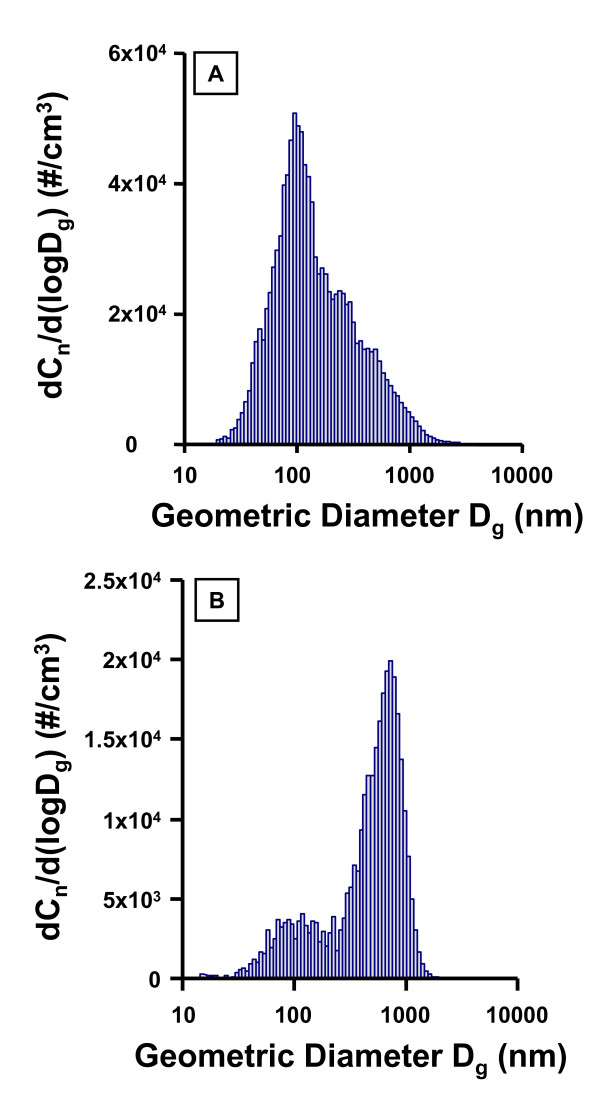
**Particle size distributions (Geometric Diameter, D_g_) of ultrafine TiO_2 _(A) and fine TiO_2 _(B) aerosols generated by the inhalation exposure system.** The ultrafine TiO_2 _aerosol has a primary mode at 100 nm and a secondary mode at 400 nm. The fine TiO_2 _aerosol has a primary mode at 710 nm and a secondary mode at 120 nm. These number-based size distributions were determined by combining the data from SMPS and APS.

Table 1 lists the exposure parameters used for the current inhalation exposures, as well as the calculated and actual deposition values. The exposure parameters were used to calculate pulmonary deposition (using aerosol concentration, minute ventilation, estimated deposition fraction, and exposure duration). The actual pulmonary deposition is used to identify experimental groups in subsequent Tables and Figures. The primary count mode for the ultrafine TiO_2 _aerosols was 100 nm, while it was 710 nm for the fine TiO_2 _aerosols (Figure 2). When considering the whole size distribution, the former had a count geometric mean diameter of 138 nm and a geometric standard deviation of 2.2, and the latter had a count geometric mean diameter of 402 nm and a geometric standard deviation of 2.4.

**Table 1:** TiO_2 _aerosol profiles and group depositions.

***Particle Type***	***Aerosol Concentration (mg/m***^3^***)***	***Exposure Time (min)***	***Calculated Deposition (μg)***	***Actual Deposition (μg)***
Sham Control	0	240	0	0
Ultrafine	10	720	150	37.60 ± 1.90
Ultrafine	12	240	60	19.17 ± 0.36
Ultrafine	6	240	30	9.48 ± 0.32
Ultrafine	3	240	15	6.38 ± 0.13
Ultrafine	1.5	240	7	3.70 ± 0.08
Fine	15	480	150	89.80 ± 6.92
Fine	16	300	100	66.50 ± 3.82
Fine	12	240	60	36.33 ± 0.69
Fine	6	240	30	19.67 ± 1.07
Fine	3	240	15	8.26 ± 0.29

**NOTE: **Analysis of the filter samples collected from the exposure chamber do not indicate any detectable quantity of copper or tin (two major components of bronze beads used in the fluidized-bed generator). This indicates that the exposure environment does not have detectable levels of contaminants from the bronze beads used to disperse the bulk TiO_2 _samples.

**Figure 3:**
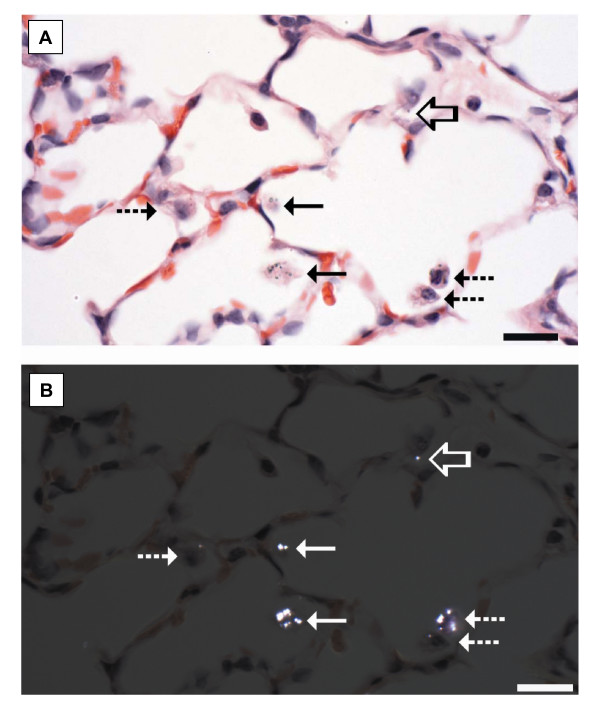
**Histopathologic alterations were subtle in the lungs of rats inhaling fine TiO_2_.** These changes principally consisted of particle accumulation. Particles were difficult to see using transmitted light (A) but were birefringent when visualized using cross-polarized light, enhancing their detection (B). The particle accumulation included apparently free TiO_2 _particles (open arrow), TiO_2 _particles within morphologically normal alveolar macrophages (dashed arrows), and TiO_2 _particles within macrophages without nuclei (anuclear macrophages, solid arrows). Both morphologically normal and anuclear macrophages were frequently in contact with the alveolar wall. H&E section. Reference bar = 20 μm.

The exposures selected for this study were exposures which do not alter bronchoalveolar lavage markers of pulmonary inflammation or lung damage. Consistent with the design, histopathology did not identify significant inflammation in lung sections. The principal histopathologic alterations in the lung consisted of particle accumulation within alveolar macrophages, the presence of anuclear macrophages, and an intimate association between particle-laden alveolar macrophages and the alveolar wall. Anuclear alveolar macrophages were not observed in any of the 21 sham-exposed rats. Anuclear alveolar macrophages were identified in 1 of 20 rats exposed to ultrafine TiO_2 _(a rat in the 38 μg exposure group), and in 16 of the 29 rats exposed to fine TiO_2 _(Figure 3A), including 1 of 6 rats in the 8 μg target exposure, 2 of 4 rats in the 20 μg target exposure, 5 of 6 rats in the 36 μg target exposure, 5 of 7 rats in the 67 μg target exposure, and 3 of 6 rats in the 90 μg target exposure. Because the macrophage cell membrane remains intact, anuclear macrophages are presumed to represent an apoptotic change.

**Figure 4:**
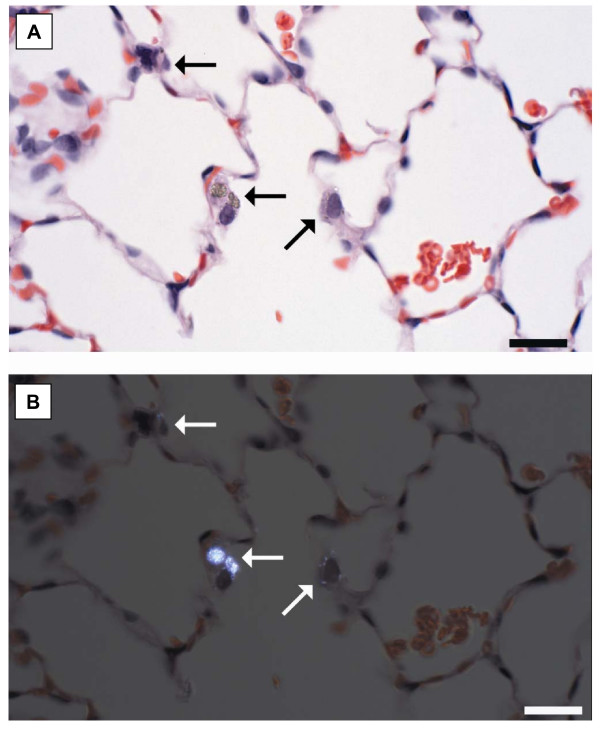
**Histopathologic alterations were particularly subtle in the lungs of rats inhaling ultrafine TiO_2_.** Ultrafine particle accumulation in alveolar macrophages (arrows) was difficult to see with transmitted light (A). Ultrafine particle accumulation within alveolar macrophages was more easily visualized as birefringent intracytoplasmic material using cross-polarized light (B). Macrophages containing ultrafine TiO_2 _particles were frequently in contact with the alveolar wall. H&E section. Reference bar = 20 μm.

In addition to the particle accumulation, macrophages containing particles were frequently intimately associated with the alveolar wall (Figures 3 and 4), a location, suggestive of, but not diagnostic of, macrophage activation. This change was difficult to quantify because it was dependent upon identifying the macrophages which were intimately associated with the alveolar wall. These macrophages were best demonstrated using cross-polarization to demonstrate the titanium particles (Figures 3B and 4B). Fine TiO_2 _particles were distinctly birefringent in cross-polarized light and could be identified in both intracellular and extracellular locations, while ultrafine TiO_2 _particles were faintly birefringent in polarized light and were only identified within macrophages (Figures 4B). Since the size of ultrafine TiO_2 _particles should be below the size visible in the light microscope, the observed ultrafine TiO_2 _particles are presumed to represent agglomerates of ultrafine particles. Consistent with the ultrafine particles only being visible when agglomerated, ultrafine particles were not observed in sham-exposed rats or rats receiving the lowest exposure, but were seen in 1 of 4 rats at 6 μg target exposure, 1 of 4 rats at the 10 μg target exposure, 2 of 4 rats at the 19 μg target exposure, and all rats receiving the 38 μg target exposure. Conversely, particles were seen in the lungs of all rats exposed to fine TiO_2 _at any dose.

At the time of intravital experiments, rats in the 38 μg ultrafine TiO_2 _group were slightly older than rats in all other experimental groups (Table 2). Despite this subtle age difference in one group, mean arterial pressure was not different among all experimental groups. Resting diameter, passive diameter and resting tone were not affected by aerosol dose, particle size or exposure method (Table 3).

**Table 2:** Profiles of experimental animals used for intravital studies.

	***Exposure Parameters***					
***TiO***_2 _***Dose/Group***	***Aerosol Concentration (mg/m***^3^***)***	***Duration (min)***	***N***	***Age (days)***	***Weight (grams)***	***Mean Arterial Pressure (mm Hg)***
Sham Control	0	240	4	43 ± 1	206 ± 11	98 ± 9
38 μg Ultrafine	10	720	3	46 ± 3 *	228 ± 29	98 ± 6
19 μg Ultrafine	12	240	5	40 ± 1	188 ± 9	95 ± 4
10 μg Ultrafine	6	240	4	42 ± 1	224 ± 7	103 ± 2
10 μg Ultrafine	3	480	5	41 ± 1	202 ± 7	102 ± 10
10 μg Ultrafine	12	120	5	42 ± 1	217 ± 6	107 ± 7
6 μg Ultrafine	3	240	3	42 ± 1	216 ± 9	93 ± 7
4 μg Ultrafine	1.5	240	3	42 ± 1	207 ± 8	96 ± 7
90 μg Fine	15	480	4	42 ± 1	233 ± 4	97 ± 2
67 μg Fine	16	300	4	41 ± 1	214 ± 11	96 ± 3
36 μg Fine	12	240	4	41 ± 1	213 ± 6	100 ± 4
20 μg Fine	6	240	3	42 ± 1	209 ± 8	101 ± 3
8 μg Fine	3	240	5	42 ± 1	233 ± 9	106 ± 6

*, P < 0.05 vs. all other experimental groups.

**Table 3:** Resting variables for all arterioles studied in intravital studies.

	***Exposure Parameters***					
***TiO***_2_***Dose/Group***	***Aerosol Concentration (mg/m***^3^***)***	***Duration (min)***	***n***	***Resting Diameter (μm)***	***Passive Diameter (μm)***	***Resting Tone (% of maximum)***
Sham Control	0	240	8	40 ± 2	99 ± 4	59 ± 3
38 μg Ultrafine	10	720	9	42 ± 2	106 ± 5	60 ± 3
19 μg Ultrafine	12	240	11	42 ± 1	100 ± 5	57 ± 3
10 μg Ultrafine	6	240	8	40 ± 2	98 ± 2	59 ± 3
10 μg Ultrafine	3	480	10	40 ± 2	95 ± 4	57 ± 3
10 μg Ultrafine	12	120	10	45 ± 2	99 ± 7	54 ± 3
6 μg Ultrafine	3	240	7	39 ± 1	105 ± 5	62 ± 2
4 μg Ultrafine	1.5	240	9	38 ± 1	97 ± 4	60 ± 2
90 μg Fine	15	480	8	45 ± 1	106 ± 5	57 ± 3
67 μg Fine	16	300	8	41 ± 1	105 ± 2	61 ± 2
36 μg Fine	12	240	8	39 ± 2	100 ± 7	60 ± 3
20 μg Fine	6	240	7	39 ± 1	92 ± 2	57 ± 2
8 μg Fine	3	240	12	41 ± 1	100 ± 5	58 ± 2

**Figure 5:**
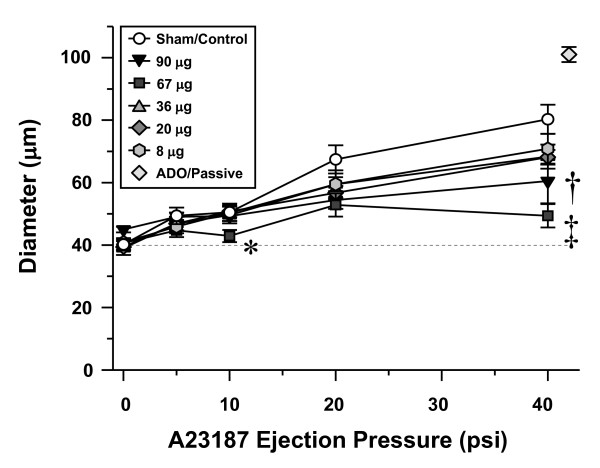
**Fine TiO_2 _inhalation impairs systemic arteriolar dilation 24 hours after exposure in a dose-dependent manner.** Sham/Control, n = 8; 90 μg, n = 8; 67 μg, n = 8; 36 μg, n = 8; 20 μg, n = 7; 8 μg, n = 12. Values are means ± SE. *, P < 0.05 vs. all groups. †, P < 0.05 vs. Sham/Control group. ‡, P < 0.05 vs. 8–36 μg groups. Adenosine (ADO).

Consistent with our previous findings in rats exposed to fine TiO_2 _via intratracheal instillation [13,14], inhalation of fine TiO_2 _impaired arteriolar dilation in response to A23187 infusion in a dose-dependent manner (Figure 5). Although significantly compromised, arteriolar dilation was still present after exposure to as much as 90 μg of fine TiO_2_. The no-effect dose was determined to be 8 μg. At this dose, arteriolar dilation at each ejection pressure was identical to that observed in the sham-control group.

**Figure 6:**
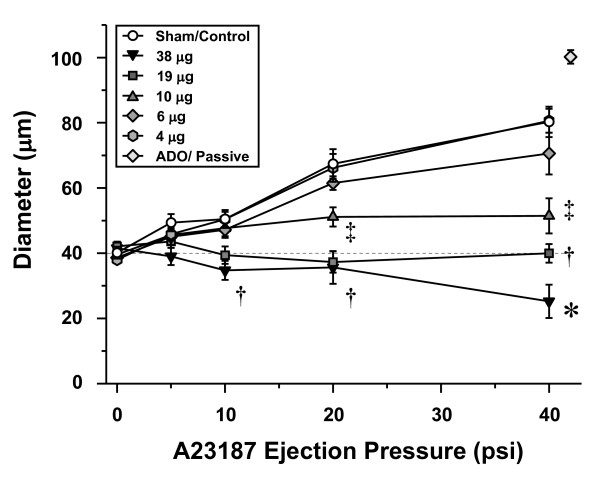
**Ultrafine TiO_2 _inhalation impairs systemic arteriolar dilation 24 hours after exposure in a dose-dependent manner.** Sham/Control, n = 8; 38 μg, n = 9; 19 μg, n = 11; 10 μg, n = 8; 6 μg, n = 7; 4 μg, n = 9. Values are means ± SE. *, P < 0.05 vs. 19 μg group. †, P < 0.05 vs. 10 μg group. ‡, P < 0.05 vs. 6 μg group. Adenosine (ADO).

Inhalation of ultrafine TiO_2 _impaired arteriolar dilation in response to A23187 infusion in a dose-dependent manner (Figure 6). This was most evident at the 38 μg dose, in which not only was dilation completely abolished, but significant arteriolar constriction resulted during A23187 infusion. The no-effect dose was determined to be 4 μg, in which arteriolar responsiveness was not different from responses observed in sham-controls.

**Figure 7:**
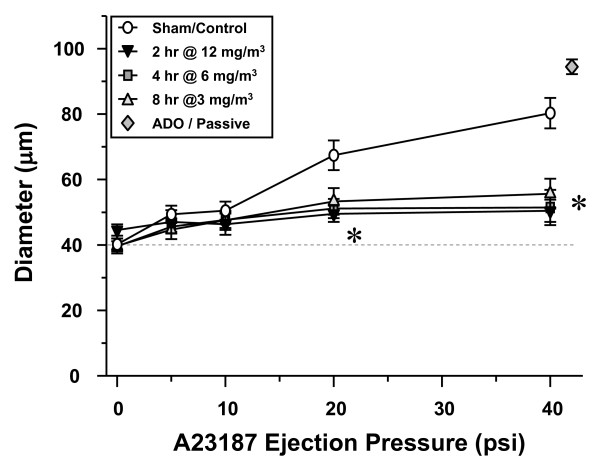
**Manipulation of ultrafine TiO_2 _inhalation exposure time or aerosol concentration does not alter the impairment of systemic arteriolar dilation.** 2 hr@12 mg/m^3^, n = 10; 4 hr@6 mg/m^3^, n = 8; 8 hr@3 mg/m^3^, n = 10. Values are means ± SE. *, P < 0.05 vs. Sham/Control group. Adenosine (ADO).

Because aerosol concentration and exposure time were manipulated to obtain an array of pulmonary depositions, the possibility existed that such subtle manipulations could themselves contribute to the resultant microvascular dysfunction. To address this possibility, additional exposures were performed in which exposure time (2–8 hrs) and aerosol concentrations (3–12 mg/m^3^) were manipulated to produce identical calculated pulmonary depositions. Three groups of rats displayed identical levels of microvascular dysfunction after exposure to 30 μg ultrafine TiO_2_via different conditions (Figure 7). This indicates that manipulation of exposure time or aerosol concentration within the parameters used in the current study neither attenuates nor augments the resultant microvascular dysfunction (i.e., the response is dependent on the time × concentration product of exposure).

**Figure 8:**
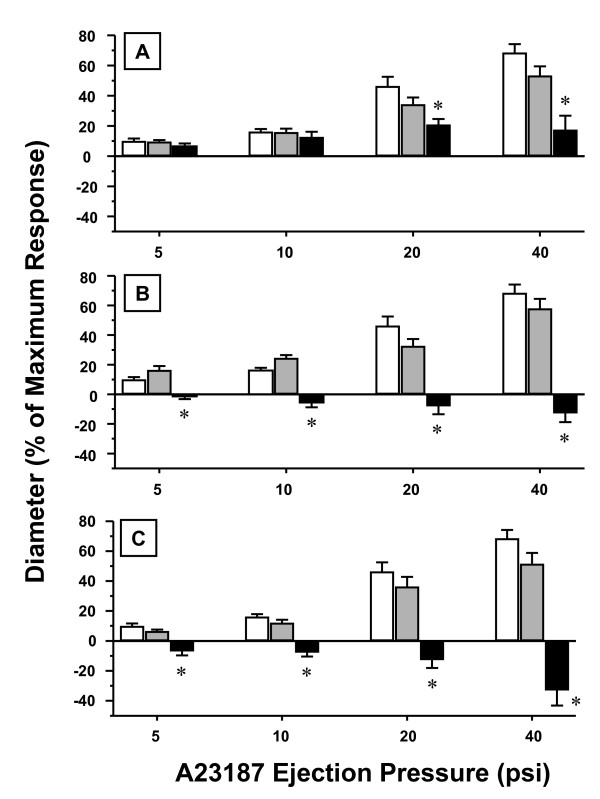
**Systemic arteriolar dilation dose-response relationships at various inhalation exposure burdens of ultrafine vs. fine TiO_2_.** Open bars in all panels, Sham/Control group. Panel A, grey bars = 8 μg fine TiO_2_; black bars = 10 μg ultrafine TiO_2_. Panel B, grey bars = 20 μg fine TiO_2_; black bars = 19 μg ultrafine TiO_2_. Panel C, grey bars = 36 μg fine TiO_2_; black bars = 38 μg ultrafine TiO_2_. *, P < 0.05 vs. Sham/Control and fine TiO_2 _at the same ejection pressure. Values are means ± SE.

Because actual pulmonary mass deposition was determined, three pair-wise comparisons of arteriolar responsiveness can be made between rats exposed to fine and ultrafine TiO_2 _(Figure 8). At a deposition of 8–10 μg, fine TiO_2 _produced no significant microvascular effects, whereas ultrafine TiO_2 _significantly impaired arteriolar dilations at 20 and 40 psi A23187 by 53% and 75%, respectively (Top Panel). At a deposition of 19–20 μg, fine TiO_2 _showed a trend towards an impaired dilation, while ultrafine TiO_2 _produced arteriolar constrictions that were significantly different from the responses in both the sham-control and fine TiO_2 _groups (Middle Panel). At a deposition of 36–38 μg, effects similar to the 19–20 μg lung burden occurred but the intensity of arteriolar constriction after exposure to ultrafine TiO_2 _was more pronounced (Bottom Panel). This suggests that at similar pulmonary burdens, ultrafine particles produce greater systemic microvascular dysfunction than fine particles.

## Discussion

This is the first study to directly identify an impaired vasodilator capacity in the systemic microcirculation after nanoparticle inhalation. A second novel observation in the current report is that for the same pulmonary mass deposition, nanoparticles produce significantly greater systemic microvascular dysfunction than their larger, fine counterparts of the same composition.

Nanoparticle inhalation attenuated systemic arteriolar dilation in a dose-dependent manner (Figure 6). This was most evident at a pulmonary deposition of 38 μg, in which arterioles constricted in response to intraluminal A23187 infusion. Arteriolar constriction during A23187 infusion is inconsistent with a healthy endothelium because under these conditions A23187 normally interacts only with endothelial cells to increase intracellular Ca^2+ ^concentration and subsequently stimulate nitric oxide production [33]. Altered endothelial integrity and/or permeability could allow luminal A23187 to interact with smooth muscle cells, thereby increasing intracellular Ca^2+ ^concentration [34] and ultimately stimulate vasoconstriction [35]. Alternatively, endothelial integrity may be unaltered, but some aspect of particle exposure offsets the prevailing balance of vasoconstrictors and vasodilators. Indeed, A23187 has the potential to stimulate endothelial production of vasoconstrictor prostanoids [36,37]. Future studies must determine if endothelial integrity has been compromised and/or the production of vasoconstrictor prostanoids has been altered by nanoparticle exposure. Of equal importance, future investigations must characterize the ability of extrapulmonary nanoparticles to alter the bioavailability of vasoactive metabolites.

The loss of microvascular vasodilator capacity can be a profound influence on normal homeostasis in any organ. In its most basic sense, this microvascular impairment would decrease tissue perfusion, and therefore, compromise function [38]. Our current techniques and findings focus upon vasomotor function in single arterioles after pulmonary nanoparticle exposure. To fully appreciate the net effect of alterations in vascular regulation, total tissue or organ blood flow must be studied. These approaches can be challenging, and as such, other hemodynamic variables that relate to tissue or organ blood flow can offer considerable insight into the systemic microvascular consequences that follow particle exposure. Mills et al. [39] showed that diesel exhaust inhalation attenuates forearm blood flow responsiveness to vasodilators. Further, pulmonary exposure to diesel particles in the ultrafine range potentiates myocardial ischemia in patients with pre-existing coronary heart disease [40]. Urch et al. [41] have reported acute increases in diastolic blood pressure within 2 hrs of particle exposure. Changes in diastolic blood pressure are achieved primarily by alterations within the resistance vasculature. Rundell et al. [42] reported a deficit in hemoglobin reoxygenation following arterial occlusion. Taken together, these findings are highly suggestive of larger disturbances in microvascular blood flow regulation after particle exposure.

Brook et al. [43] initially identified a subtle vasoconstriction of the brachial artery in humans that inhaled fine particle matter. Recently, Rundell et al. [42] reported that this conduit artery constricts similarly after ultrafine particle inhalation. Because the smaller particles used by Rundell et al. did not appear to be associated with a more robust vasoconstriction in these studies (than those used by Brook et al.), one might conclude that particle size does not dictate resultant vascular dysfunction. However, it is possible that this experimental model lacks sufficient sensitivity to reveal such differences, or that fundamental differences in particle composition, exposure protocols, and/or experimental group profiles prevent a meaningful comparison between the two studies.

Ultrafine TiO_2 _has been shown to cause significantly greater airway inflammation than fine TiO_2 _[5]. Based upon the count geometric mean diameter (Figure 2), nano-sized titania aerosols attenuate systemic endothelium-dependent arteriolar dilation to a greater degree than fine TiO_2 _aerosols (Figures 5, 6 and 8) at similar lung burdens (Table 1). This observation is most evident at lung burdens of 36 μg and 38 μg for fine and ultrafine particles, respectively. In this case, nano-sized titania aerosol exposure was consistently associated with arteriolar constriction, whereas fine TiO_2 _inhalation was consistently associated with an impaired arteriolar dilation (Figure 8, bottom panel). This suggests that given the diverse nature of particle size, shape and chemistry, the deposited particle mass may not be the ideal dose metric.

Data presented in the current study indicate that on an equivalent mass basis, ultrafine TiO_2 _was approximately one order of magnitude more potent than fine TiO_2 _in causing systemic microvascular dysfunction. Oberdorster [44] has proposed that particle surface area may be the more appropriate dose metric for pulmonary effects of ultrafine particles than mass. Therefore, we also analyzed the results on an equivalent surface area of particles deposited in the lungs. BET analysis, as described by Brunauer, Emmett and Teller [45], was used to determine that the surface area of ultrafine TiO_2 _was 48.08 m^2^/g while fine TiO_2 _was 2.34 m^2^/g; i.e., ultrafine TiO_2 _had approximately 20 times more surface area per unit mass than fine TiO_2_. Therefore, if one normalized the systemic microvascular response to equivalent total particle surface area, the fine TiO_2 _would be more potent than the ultrafine TiO_2_. This conclusion is likely the result of an over estimation of the total ultrafine surface area delivered to the lungs since the BET method measures the gas absorptive surface area of the primary particles, rather than the actual physical surface of the aerosolized agglomerates. Indeed, Shvedova et al. [46] reported that the pulmonary response to ultrafine carbon black was significantly increased upon improved nanoparticle dispersion.

It is important to note that actual pulmonary particle deposition was significantly less than the calculated deposition (Table 1). This may be due to a number of reasons. First, it is very likely that particle clearance occurs in the 24 hrs after exposure, and particularly during the 720 minute exposures that took place over three days. Second, biological heterogeneity among rats may contribute to differences in the calculated vs. the actual particle depositions. In this case, subtle differences in minute volumes or deposition fractions among rats could contribute to the divergent measurements. However, despite manipulation of aerosol concentration (3–12 mg/m^3^) or exposure time (2–8 hrs), the intensity of microvascular dysfunction was not different among these groups 24 hrs after exposure (Figure 7). This suggests that the ranges of variables used to obtain our target pulmonary loads are not responsible for observed biologic effects in either the lung or the systemic microcirculation.

The opportunity to directly compare the microvascular responses to two different exposure methods presented itself in this study. When arteriolar responses between rats exposed to similar doses of fine TiO_2 _(90 μg via inhalation vs. 100 μg via intratracheal instillation [14]) are compared, no significant differences are apparent in the resultant microvascular dysfunction (data not shown). This suggests that the method of particle introduction to the lungs does not artificially induce systemic microvascular dysfunction.

The physiological relevance of intratracheal instillation is frequently questioned. Intratracheal instillation is a commonly used exposure method because it is rapid, economical and consistent across multiple animals. Further, intratracheal instillation can produce a relatively uniform pulmonary particle distribution if exposure doses are kept low [47]. However, in regards to the true in vivo environment, intratracheal instillation bypasses many important physiological processes. Hence, the "gold standard" of particle exposure remains via inhalation.

## Conclusion

In the time since we first reported that systemic microvascular dysfunction follows PM exposure [13], nanotechnology products have become considerably imbedded in most every aspect of daily life. It is also clearly apparent now that the benefits of nanotechnology are far reaching. Unfortunately, not only are the precise mechanisms through which inhaled particles exert systemic biologic effects unknown, but also the scope of biologic targets and/or end points is greater when nanoparticles are considered. By definition, this later observation may be due largely because of particle size, but surface chemistry of nanoparticles must also be taken into account in future studies. The ultrafine particles used in the current study represent manufactured particles in the "nano" size range, and after pulmonary deposition, they exerted a robust biologic effect. It is unclear at this time what the most appropriate dose metric is for nanoparticles, but it is apparent from our studies and many others that mass does not consistently appear to be the ideal metric. Independent of the appropriate dose metric is our consistent observation that pulmonary particle exposure initiates systemic microvascular dysfunction, and this observation is now extended to nanoparticles.

## List of abbreviations

A23187 – Calcium ionophore. ADO – Adenosine. ANOVA – Analysis of variance. ICP-AES – Inductively-coupled plasma-atomic emission spectroscopy. D_c _– Control diameter. D_pass _– Passive diameter. D_ss _– Steady state diameter. i.p. – Intraperitoneal. N – Number of animals. n – Number of arterioles. PM – Particulate matter. TiO_2 _– Titanium dioxide

## Competing interests

The author(s) declare that they have no competing interests.

## Authors' contributions

TRN performed intravital microscopy experiments. DWP performed ICP-AES experiments. AFH performed pulmonary histology. JLC, BTC and DGF performed inhalation exposures. TRN and VC conceived and designed the study. All authors read and approved the final manuscript.

## Disclaimers

The findings and conclusions in this report are those of the authors and do not necessarily represent the views of the National Institute for Occupational Safety and Health.

Research described in this article was conducted under contract to the Health Effects Institute (HEI), an organization jointly funded by the United States Environmental Protection Agency (EPA) (Assistance Award No. R-82811201) and certain motor vehicle and engine manufacturers. The contents of this article do not necessarily reflect the views of HEI, or its sponsors, nor do they necessarily reflect the views and policies of the EPA or motor vehicle and engine manufacturers.

## Support

Health Effects Institute Award #4730 and NIEHS ES015022 (TRN).
